# Sphingosine 1-Phoshpate Receptors are Located in Synapses and Control Spontaneous Activity of Mouse Neurons in Culture

**DOI:** 10.1007/s11064-022-03664-3

**Published:** 2022-07-04

**Authors:** Cecilia Skoug, Isak Martinsson, Gunnar K. Gouras, Anja Meissner, João M. N. Duarte

**Affiliations:** 1 Department of Experimental Medical Science, Faculty of Medicine, Lund University, Lund, Sweden; 2 Wallenberg Centre for Molecular Medicine, Lund University, Lund, Sweden; 3 Experimental Dementia Research Unit, Lund University, Lund, Sweden; 4 Department of Physiology, University of Augsburg, Augsburg, Germany

**Keywords:** Primary neurons, Calcium imaging, Synaptic fractioning, S1P, Sphingolipids

## Abstract

Sphingosine-1-phosphate (S1P) is best known for its roles as vascular and immune regulator. Besides, it is also present in the central nervous system (CNS) where it can act as neuromodulator via five S1P receptors (S1PRs), and thus control neurotransmitter release. The distribution of S1PRs in the active zone and postsynaptic density of CNS synapses remains unknown. In the current study, we investigated the localization of S1PR1-5 in synapses of the mouse cortex. Cortical nerve terminals purified in a sucrose gradient were endowed with all five S1PRs. Further subcellular fractionation of cortical nerve terminals revealed S1PR2 and S1PR4 immunoreactivity in the active zone of presynaptic nerve terminals. Interestingly, only S1PR2 and S1PR3 immunoreactivity was found in the postsynaptic density. All receptors were present outside the active zone of nerve terminals. Neurons in the mouse cortex and primary neurons in culture showed immunoreactivity against all five S1PRs, and Ca^2+^ imaging revealed that S1P inhibits spontaneous neuronal activity in a dose-dependent fashion. When testing selective agonists for each of the receptors, we found that only S1PR1, S1PR2 and S1PR4 control spontaneous neuronal activity. We conclude that S1PR2 and S1PR4 are located in the active zone of nerve terminals and inhibit neuronal activity. Future studies need to test whether these receptors modulate stimulation-induced neurotransmitter release.

## Introduction

Sphingolipids play important biological roles, and defects in metabolism of sphingolipids have been linked to neurodegenerative diseases, such as Alzheimer’s disease, Parkinson's disease and multiple sclerosis [1]. Particularly interesting is sphingosine-1-phosphate (S1P) and its signaling axis, which have been implicated in mechanisms of neurodegeneration and neuroinflammation [2–5] and thus, have been proposed to offer neuroprotection targets [1].

S1P is a lysophospholipid with pleiotropic functions that are mediated both via intracellular signaling and by binding to five G protein-coupled membrane S1P receptors S1PR1-5 [6, 7]. Their subsequent signaling through G_i_, G_q_ or G_12/13_ relays signals to phosphoinositade 3-kinase (PI3K), protein kinase C (PKC), phospholipases or cyclic adenosine monophosphate (cAMP) [8]. All S1PRs have been identified in at least one cell type of the central nervous system (CNS), such as neuroblasts, neurons, astrocytes, microglia and oligodendrocytes (reviewed by Groves et al. [9]). S1PRs influence important functions in CNS development and further have roles in pathological conditions, such as brain ischemic stroke, schizophrenia and multiple sclerosis, as well as hearing loss and seizures [7]. S1PR1 is important during development, namely for vascularization and neurogenesis [10], and has a key role in immunological development as it regulates the amount of circulating immune cells [11]. S1PR2 plays an essential role in regulating endothelial polarity, which influences blood–brain-barrier permeability and capture of infiltrating lymphocytes as it is connected to regulation of adherent junctions [12]. S1PR2 is also functional in neurons, and its genetic deletion in mice induces hyper-excitability [13]. S1PR3 is present in astrocytes and microglia, and is involved in gliosis and neuroinflammation processes [14–17]. Although little importance has been given to brain S1PR4 [18], it is expressed during neuronal development [19], and it can modulate brain function through regulating blood–brain-barrier permeability [20]. S1PR5 is considered to be mostly located within oligodendrocyte precursor cells and mature oligodendrocytes, where it contributes to regulate the differentiation process [21].

As sphingolipids and their metabolites are reported to modulate multiple aspects of cellular survival, stress response and aging, the significance of studying these interactions in the CNS in relation to neurodegenerative diseases and aging could provide opportunities for therapeutic interventions. Fingolimod (FTY720; approved for the treatment of remitting relapsing multiple sclerosis) is a S1P receptor modulator that crosses the blood brain barrier, is phosphorylated and acts on central S1PRs [9, 22]. Fingolimod interacts with S1PR1,3,4,5, and has been shown to provide neuroprotection in animal models of brain ischemia [23–25], traumatic brain injury [25], epilepsy [26, 27], Parkinson's disease [28, 29], and Alzheimer's disease [30–32]. Interestingly, lower S1P levels and sphingosine kinase activity were reported to occur early in Alzheimer’s disease pathogenesis and thus, constitute a possible target for preventing disease development [33].

Neuromodulation roles of S1P are not fully understood. It has been suggested that the unspecific S1P modulator Fingolimod inhibits glutamate release from synaptosomes [34], and promotes neurogenesis and neuronal survival [35]. These actions are attributed to the binding to S1PR1, but other S1PRs in neurons might contribute to neuronal Fingolimod effects. In this study, we aimed at determining the synaptic distribution of S1PRs, and explored their ability to control neuronal activity.

## Materials and Methods

### Drugs

S1P was obtained from Cayman chemicals (BioNordika, Sweden). Selective agonists for S1PR1-5 were, respectively, 5-[4-Phenyl-5-(trifluoromethyl)thiophen-2-yl]-3-[3-(trifluoromethyl)phenyl]1,2,4-oxadiazole (≥ 99%, SEW2871 from Tocris Bio-Techne, Abingdon, UK), 1-[2-[2,5-Dimethyl-1-(phenylmethyl)-1*H*-pyrrol-3-yl]-2-oxoethyl]-1,6-dihydro-6-oxo-3-pyridinecarbonitrile (≥ 99%, CYM5520, Tocris), *N,N*-Dicyclohexyl-5-cyclopropyl-3-isoxazolecarboxamide (≥ 97%, CYM5541, Tocris), (2Z,5Z)-5-[[1-(2,4-Difluorophenyl)-2,5-dimethyl-1*H-*pyrrol-3-yl]methylene]-2-[(2-methoxyethyl)imino]-3-methyl-4-thiazolidinone (≥ 98%, CYM 50308, Tocris), 1-[[4-[(3,4-Dichlorophenyl)methoxy]phenyl]methyl]-3-azetidinecarboxylic acid (≥ 99%, A971432, Tocris). Fatty-acid free bovine serum albumin (BSA; Sigma-Aldrich) was used to prepare the vehicle for S1P delivery. Polyethyleneglycol of molecular weight 190–210 (PEG; Sigma-Aldrich #88440) was used as vehicle for S1PR agonists.

### Animals

Experiments were performed according to EU Directive 2010/63/EU under approval of the Malmö/Lund Committee for Animal Experiment Ethics, and are reported following the ARRIVE guidelines (Animal Research: Reporting In Vivo Experiments, NC3Rs initiative, UK). Sample size was estimated from previous work [36]. Male and female C57BL/6 J mice were obtained from Taconic Biosciences (Ry, Denmark). Mice were housed in groups of 4–5 animals on a 12 h light–dark cycle with lights on at 07:00, room temperature of 21–23 °C and humidity at 55–60%. Mice were habituated to the facility for at least 1 week before experimentation. Food and water were provided ad libitum.

### Brain Tissue Sampling

Six mice (10–14 weeks old) were anaesthetized with isoflurane and quickly decapitated, and brains were dissected. For preparation of synaptosomes brains were kept in isolation buffer (see below) on ice. For total protein extracts, brain samples were frozen in N_2_ (l) and stored at − 80 °C until further experiments.

### Preparation of Synaptosomes and Synaptic Fractions

Synaptosomal fractionation was modified from Morato et al. [37]. Briefly, the mouse cortex was homogenized in 1 mL of isolation buffer (in mmol/L: 320 sucrose, 0.1 CaCl_2_, 0.1 MgCl_2_, pH 7.4) at 4 °C in a 5-mL Potter–Elvehjem glass/teflon homogenizer (10 strokes at 700–900 rpm). The resulting homogenate was mixed with 6 mL sucrose (2 mol/L) and 2.5 mL CaCl_2_ (0.1 mmol/L) in an ultra-clear centrifuge tube (#344059, Beckman Coulter, USA). Then, 2.5 mL of sucrose (1 mol/L) containing 0.1 mM CaCl_2_ was carefully added on top to form a discontinuous sucrose gradient. All centrifugations were performed in an Optima XL-100 K Ultracentrifuge (Beckman Coulter) with SW41Ti swinging bucket rotor (Beckman Coulter). After centrifugation for 3 h at 100,000 g, 4 °C, the synaptosomes were collected from the interphase between 1.25 and 1 mol/L sucrose and diluted 10 times in isolation buffer, centrifuged for 30 min at 15,000 g, 4 °C, and the resulting synaptosomal pellet was re-suspended in 1 mL of isolation buffer.

For fractioning synaptosomes, part of each sample was diluted 1:5 in 0.1 mmol/L CaCl_2_, and an equal volume of solubilization buffer (2% Triton X-100, 40 mmol/L Tris, pH 6.0) was added to the suspension. The suspension was incubated for 30 min on ice with constant agitation and the insoluble material (synaptic junctions) was pelleted by centrifugation for 30 min at 40,000 g, 4 °C. The supernatant (extra-synaptic fraction) was concentrated using an Amicon Ultra 15 10 K (#UFC901008, Merck Millipore, Ireland) and protein was precipitated with six volumes of acetone at – 20 °C and recovered by centrifugation for 30 min at 18,000 g, – 15 °C. The pellet containing synaptic junctions was washed in solubilization buffer at pH 6.0, and then re-suspended in 10 volumes of a second solubilization buffer (1% Triton X-100 and 20 mmol/L Tris, pH 8.0). After incubation under agitation for 30 min on ice, the mixture was centrifuged and the supernatant (pre-synaptic fraction) was processed as described for the extra-synaptic fraction, whereas the insoluble pellet corresponds to the post-synaptic fraction All synaptic fractions were re-suspended in 5%(w/v) sodium dodecylsulfate with protease inhibitors (#11697498001, Roche, Switzerland).

### Total Protein Extracts

Tissue samples were homogenized with a sonicator probe in lysis buffer [in mmol/L: 150 NaCl, 1 EDTA, 50 tris(hydroxymethyl)aminomethane (Tris)-HCl, 1% (w/v) sodium dodecylsulfate, pH 8.0] containing protease inhibitors and phosphatase inhibitors (#4906837001, Roche, Switzerland). The homogenate was maintained in constant agitation for 2 h at 4 °C. After centrifugation at 3000 g for 10 min at 4 °C to remove major debris, the supernatant was saved.

### Immunoblotting

Total protein content of the samples was measured with the bicinchoninic acid assay (kit from Pierce, Thermofisher Scientific, Göteborg, Sweden). Then, Western blotting was carried out as detailed previously [38]. Briefly, samples were heated for 5 min at 95 °C in sample buffer (#NP0007, Invitrogen, USA), and then separated on 4–12% Bis–Tris mini gels (#NP0336, Invitrogen, USA), followed by transfer onto nitrocellulose membranes, pore size 0.45 μm (#GE10600002, GE Healthcare, Germany). The membranes were blocked for 60–120 min in 5% milk or bovine serum albumin in Tris-buffered saline (in mmol/L: 20 Tris, 150 NaCl, pH 7.6) containing 1% Tween 20, and incubated with primary and secondary antibodies (Table 1) diluted in this blocking solution. Immunoblots were developed with a chemiluminescence kit (#34580, Thermofisher, USA) using the Chemidoc XRS + interfaced to Image Lab 5.2.1 for image analysis (Biorad, Stockholm, Sweden).

**Table 1:** Primary antibodies used for Western blot (WB) and immunofluorescence microscopy in neurons (IFn) or brain slices (IFs)

Antibody	Dilution	Source
Rabbit anti-S1PR1	1:1000 (WB)/1:500 (IFn)/1:200 (IFs)	Thermofisher (PA1-40,000)
Rabbit anti-S1PR2	1:500 (WB)/1:250 (IFn)/1:100 (IFs)	Origene (AP01311PU-N)
Rabbit anti-S1PR3	1:1000 (WB)/1:500 (IFn)/1:200 (IFs)	Origene (TA329055)
Rabbit anti-S1PR4	1:1000 (WB)/1:250 (IFn)/1:100 (IFs)	Novus (NBP2-24,500)
Rabbit anti-S1PR5	1:500 (WB)/1:250 (IFn)/1:100 (IFs)	Novus (NBP2-24,712)
Rabbit anti-PSD95	1:2000 (WB)	Abcam (ab76115)
Rabbit anti-SNAP25	1:5000 (WB)	Abcam (ab109105)
Rabbit anti-synaptophysin	1:10,000 (WB)	Abcam (ab32127)
Chicken anti-MAP2	1:500 (IFn)	Abcam (ab5392)
Goat anti-GFAP	1:500 (IFs)	Abcam (ab53554)
AlexaFlour488-tagged rabbit anti-MAP2	1:500 (IFs)	Abcam (ab225316)
HRP-tagged anti-rabbit IgG	1:5000 (WB)	Abcam (ab6802)
AlexaFluor405-conjugated anti-goat IgG	1:1000 (IFs)	Invitrogen (ab175664)
AlexaFluor488-conjugated anti-rabbit IgG	1:1000 (IFn)	Invitrogen (R37116)
AlexaFlour568-conjugated anti-rabbit IgG	1:1000 (IFs)	Invitrogen (A21069)
AlexaFluor647-conjugated anti-chicken IgY	1:1000 (IFn)	Abcam (ab150171)

### Neuronal Cell Cultures

Primary embryonic neurons were prepared from the cortices and hippocampi of embryonic day 15–17 WT mouse embryos, as detailed by [36]. Briefly, neurons were dissociated through trypsinization and subsequent trituration in Dulbecco’s modified Eagle medium (DMEM, 30243.01#, Cytiva, Marlborough, MA-USA) supplemented with 10% fetal bovine serum (#10100-147, Gibco, Australia), 1% penicillin–streptomycin (#15140122, Thermofisher) and then placed onto poly-D-lysine coated coverslips. After 3–5 h, medium was switched to Neurobasal medium supplemented with glutamine (#25030081, Thermofisher), B27 (#A3582801, Thermofisher) and penicillin–streptomycin. Cells were cultured in vitro for 19–21 days before being used for experiments.

### Spontaneous Neuronal Activity by Ca^2+^ Imaging

Before imaging, primary neurons were incubated for 30 min with 2 µmol/L of the green-fluorescent calcium indicator Fluo-4 (#F14201, Invitrogen, Thermofisher) prepared in 0.2%(v/v) DMSO. Live-cell microscopy was performed with a Nikon Eclipse Ti microscope at 10 × with 1.4 NA. Live cell imaging chamber (Okolab, Pozzuoli, Italy) was kept at 5% CO_2_ and 37 °C. Cells were imaged every 100 ms for a duration of 2 min with an iXon Ultra CCD camera (ANDOR Technology, Belfast, UK).

S1P (Avanti Lipids) stock was prepared in 4% fatty acid-free BSA. S1P was at concentrations of 1, 10, 100 or 1000 nmol/L or vehicle (4% fatty acid-free BSA in water) was added to the cells 1 min prior to imaging. S1PR agonists (Table 2; stock solution prepared in 5% PEG in water) or vehicle (5% PEG in water) were added to the cells 1 min prior to imaging. For verifying that the experimental system detects changes in neuronal activity (hyper-/hypo-activity), cells were exposed to 20 μmol/L bicuculline methbromide (Sigma-Aldrich) or 1 μmol/L tetrodotoxin citrate (Tocris) for 15 min prior to imaging. All experiments were conducted in a paired fashion, that is, neurons prepared from an embryo received vehicle and all S1P doses, or vehicle and S1PR agonists.

**Table 2:** S1PR agonists, EC50 and concentration applied to cultured neurons in this study

	Drug	Pubchem ID	Reported EC50 (nmol/L)	Concentration (nmol/L)
S1PR1 agonist	SEW2871	4077460	14–21 (Sanna et al. [59])	150
S1PR2 agonist	CYM5520	25110470	480 (Satsu et al. [60])	150
S1PR3 agonist	CYM5541	17253208	72–132 (Jo et al. [61])	75
S1PR4 agonist	CYM50308	49835928	56 (Urbano et al. [62])	75
S1PR5 agonist	A971432	46872626	4–6 (Hobson et al. [63])	25

Regions of interest (ROIs) were defined for the soma of neurons in each stack of Ca^2+^ images using ImageJ (NIH, Bethesda, MD, USA). Fluorescence intensity over time was extracted, baseline corrected and normalized, and analyzed in Peakcaller [39] running on MATLAB 2019a (MathWorks, Natick, MA-USA). Peak amplitude was calculated as fluorescence variation and normalized to baseline fluorescence (ΔF/F). Spike detection threshold was set to 3% above baseline. Non-responding cells were considered silent neurons and excluded for analysis of spiking frequency. Cells with non-neuronal shape and with full-width at half-maximum (FWHM) above 50 ms were not analyzed. Cells showing only one spike were excluded from frequency analysis, as their frequency becomes the inverse of the imaging period.

### Immunofluorescence Confocal Microscopy

Primary neurons on cover glasses were fixed for 15 min in 4%(w/v) paraformaldehyde and 4%(w/v) sucrose at room temperature, and stored in phosphate-buffered saline (PBS; in mmol/L: 137 NaCl, 2.7 KCl, 1.5 KH_2_PO_4_, 8.1 Na_2_HPO_4_, pH 7.4) at 4 °C until usage. Fixed neurons or 20-µm cryostat-sectioned brain slices (prepared as reported by Garcia-Serrano et al. [40]) were incubated for 1 h at room temperature with blocking buffer [PBS containing 5% (v/v) goat serum (#16210-064, Gibco, New Zealand), 1% (w/v) bovine serum albumin and 0.3% (v/v) Triton X-100], followed by 2-h incubation with primary antibodies (Table 1). After washing in PBS, the samples were incubated with AlexaFluor-conjugated secondary antibodies, washed again, mounted for microscopy with ProLong Glass Antifade (#P36980, Invitrogen, USA), and examined under a Nikon A1RHD confocal microscope with a CFI Apochromat TIRF 100 × Oil, NA 1.49 or CFI Plan Apochromat Lambda 20×/40× NA 0.75 (Nikon Instruments, Tokyo, Japan). Images were acquired with NIS-elements (Laboratory Imaging, Nikon), and then processed in ImageJ.

### Statistics

Results are presented as mean ± SD of independent biological replicates (mice/embryos). Data were analyzed by paired Student t-tests (for Ca^2+^ imaging data), or by repeated-measures ANOVA (for immunoblotting data) followed by independent comparisons with the Fisher’s least significant difference (LSD) test in Prism 9.3.1 (GraphPad, San Diego, CA-US; RRID:SCR_002798). Dose-dependent effects of S1P were fitted to an inverted logistic sigmoid function to determine the IC50 (concentration that provokes a response half way between the maximal response and the maximally inhibited response).

## Results

**Fig. 1:**
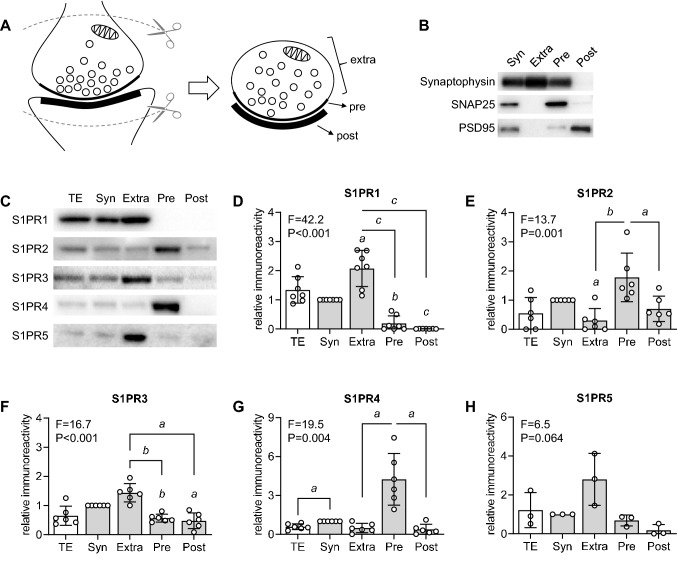
S1PRs are present in synapses of the mouse cortex. **A** Schema depicting synaptosome preparation that is then fractioned into pre-synaptic (Pre), post-synaptic (Post) and extra-synaptic (Extra) fractions. **B** Immunoblotting of synaptic fractions prepared from the mouse cortex show enrichment in SNAP25, PSD95 and synaptophysin in pre, post and extra, respectively. **C** Typical relative immunoreactivity against S1PR1-5 in total protein extracts (TE), synaptosomes (Syn), extra-, pre- and post-synaptic fractions. Protein loaded in gels was 20 µg. **D**–**H** Quantitative analysis of Western blot immunoreactivity against S1PR1-5. Circles represent individual replicates and bars are mean ± SD of n = 3–6. ANOVA F- and P-values comparing protein extracts are shown within each graph. Letters over data-points indicate significant differences relative to immunoreactivity in synaptosomes or as indicated (^*a*^P < 0.05, ^*b*^P < 0.01 and ^*c*^P < 0.001) based on Fisher’s LSD post hoc comparison after repeated-measures one-way ANOVA

**Fig. 2:**
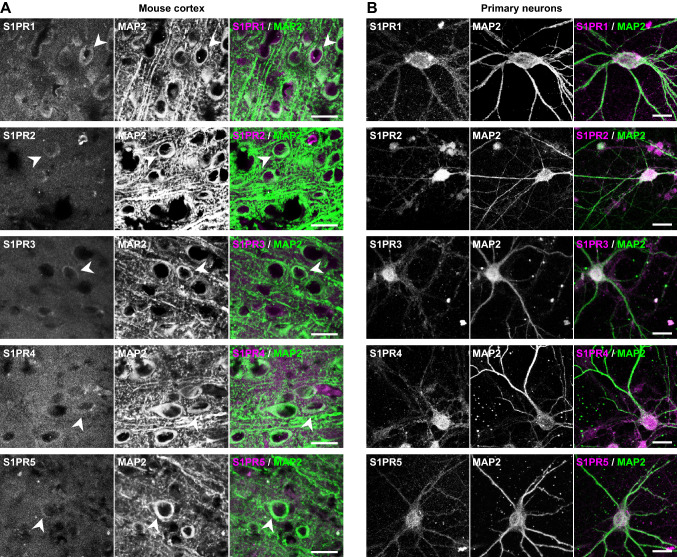
Identification of S1PRs in neurons of the mouse cortex (**A**), and in primary cultured neurons (**B**). Representative immunofluorescence micrographs of mouse brain sections and cultured cortical neurons show staining for S1PR1-5 (magenta) and the neuronal marker MAP2 (green). S1PR1-5 immunoreactivity was identified in MAP2^+^ cells in the cortex (examples indicated by arrows), and throughout the cell soma and axons of cultured neurons. Scale bars in micrographs are 20 µm

Synaptosomes from the mouse cortex were subsequently fractioned into synaptic preparations that are rich in PSD95 (post-synaptic density protein 95), SNAP25 (synaptosomal-associated protein 25) and synaptophysin, which correspond to the post-, pre- and extra-synaptic zones, respectively (Fig. 1A). Immunoreactivity against all five S1PRs was observed in synapses (Fig. 1B). Immunoreactivity quantification reveals larger S1PR1 and S1PR3 enrichment in the extrasynaptic fraction than in pre- and postsynaptic fractions (Fig. 1C), while S1PR2 and S1PR4 immunoreactivity is strongest in presynaptic fractions. Furthermore, S1PR4 was more enriched in synapses than in total protein extracts. Immunofluorescence microscopy also confirmed the presence of S1PR1-5 in cortical neurons (Fig. 2A). While immunoreactivity for some S1PRs was observed nearly in all MAP2^+^ cells (neurons), that was not the case for S1PR5, which immunoreactivity was only found in a few neurons of the mouse cortex.

**Fig. 3:**
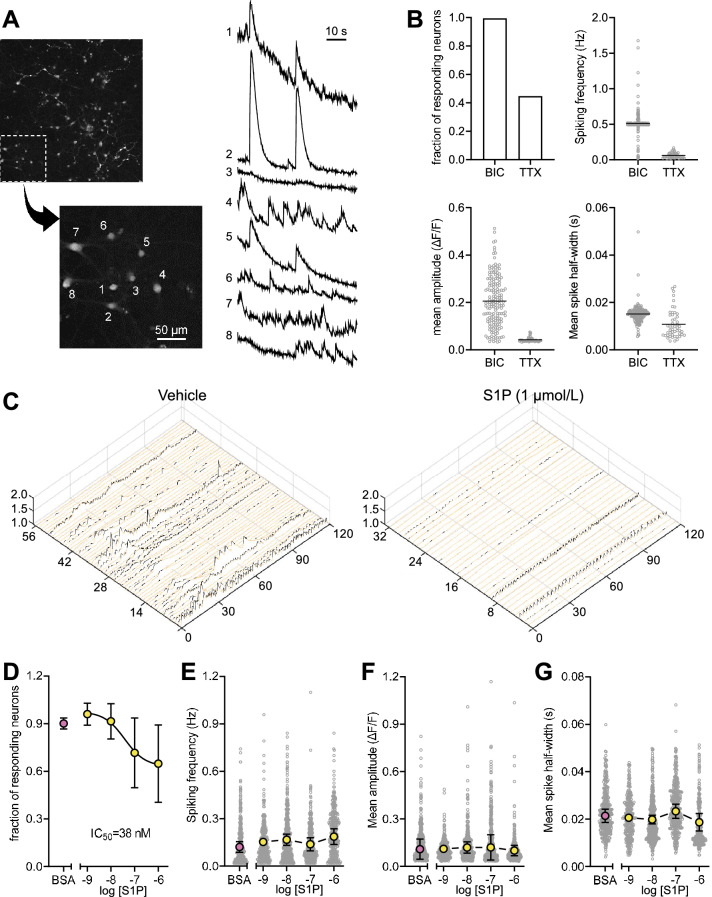
Dose response of S1P on neuronal activity measured by live Ca^2+^ imaging. **A** Examples of raw fluorescence Ca^2+^ signals traced with fluo-4 from 8 nearby cells identified as neurons. The scale bar in the micrograph represents 50 μm. **B** Control experiments depicting effect of bicuculline (BIC, 20 μmol/L) and tetrodotoxin (TTX, 1 μmol/L) on the number of responding neurons, spiking frequency, peak amplitude and half-width calculated at the half-maximum amplitude of the peak. Symbols represent each neuron analysed in 3 experiments from one cell isolation (1 biological replicate). **C** Typical fluorescence Ca^2+^ traces in neurons exposed to 1 µmol/L S1P or vehicle (BSA) as processed in PeakCaller. Time in seconds, neuron number and signal amplitude (ΔF/F) are in x- y- and z-axis, respectively. **D** S1P (concentrations in mol/L) reduced the number of responding neurons in a dose dependent manner with IC_50_ of 38 nmol/L, but had no overall effect on spiking frequency (**E**), peak amplitude (**F**) or half-width calculated at the half-maximum amplitude of the peak (**G**). Data in D-G shows mean ± SD of 3 biological replicates overlaid, when applicable, on top of smaller circles representing single neuron measurements

We next investigated whether S1PRs are present and functionally active in cultured neurons. Immunofluorescence microscopy showed the presence of all five S1PRs in primary neurons in culture (Fig. 2B). Live cell imaging with the Ca^2+^ indicator Fluo-4 was used to investigate spontaneous activity of neurons in culture. This primary culture of mixed cortical and hippocampal neurons displayed a wide range of activity patterns (Fig. 3A), and acute S1P administration inhibited neuronal activity (Fig. 3B). Namely, S1P reduced the number of responding neurons in a dose-dependent manner with IC_50_ of 38 nmol/L (Fig. 3C), while having negligible effects on spiking frequency, amplitude or FWHM (Fig. 3D–F).

**Fig. 4:**
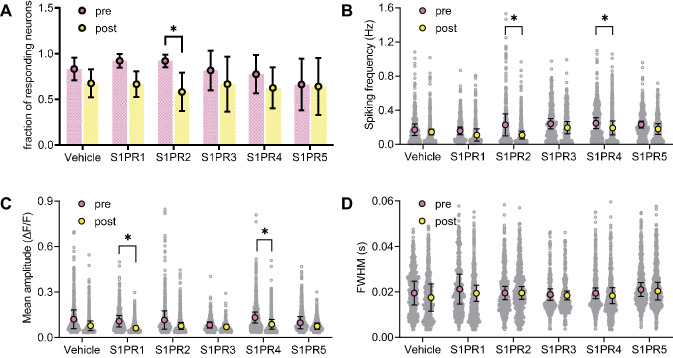
Effect of acute S1PR activation on spontaneous activity of cortical neurons in culture, as depicted by changes in number of responding cells (**A**), spiking frequency (**B**), peak amplitude (**C**) and full-width at half-maximum (FWHM) of the peak (**D**). Imaging was conducted before (pre) and after (post) administration of S1PR agonists or vehicle. Data is shown as mean ± SD of n = 4–6 overlaid on top of, when applicable, smaller circles representing single neuron measurements. Symbols over data-points indicate significant differences between pre and post (*P < 0.05) based on paired Student t-tests

To test S1PR involvement in controlling spontaneous activity of cultured neurons, calcium transients were measured after applying selective S1PR agonists. Activation of S1PR2 with the selective agonist CYM5520 (150 nmol/L) reduced the fraction of responding neurons (P < 0.05, Fig. 4A). Neuronal spiking frequency was reduced by the S1PR2 agonist, and by activation of S1PR4 by CYM50308 (75 nmol/L) (P < 0.05 for both, Fig. 4B). The mean amplitude of neuronal spikes was reduced by activation of S1PR1 with the selective agonist SEW2871 (150 nmol/L) and of S1PR4 with the selective agonist CYM50308 (75 nmol/L) (P < 0.05 for both, Fig. 4C). Selective activation of S1PR3 with 75 nmol/L CYM5541, or of S1PR5 with 25 nmol/L A971432 did not significantly impact neuronal activity. FWMH was not affected by any S1PR agonist (Fig. 4D).

## Discussion

This study demonstrates that mouse cortical neurons and their synapses are endowed with all five S1PRs, and that S1PR2 and S1PR4 are particularly enriched in the active zone of the presynaptic nerve terminal. Furthermore, calcium imaging reveals that spontaneous activity of primary neurons in culture is inhibited by S1P, which is not surprising since it has been reported that Fingolimod inhibits glutamate release [34]. The present study, however, demonstrates that selective agonists of S1PR1, 2 and 4, but not of S1PR3 or 5, can inhibit either the frequency or amplitude of spontaneous calcium spikes in primary neurons.

We observed immunoreactivity against all S1PRs in neurons, and in synapses, which is in line with RNA sequencing data for both human and murine brain cells showing expression of all S1PRs in neurons, as well as astrocytes [41]. In contrast, earlier studies have reported that primary rat hippocampal neurons only express mRNA for S1PR1 and S1PR3 [42]. To specifically localize S1PR expression in synapses, we prepared subcellular fractions of cortical nerve terminals and found all five S1PRs expressed outside the active zone of nerve terminals. In addition to that, S1PR2 and S1PR4 immunoreactivity was also observed in the active zone of presynaptic nerve terminals, which spurs the speculation of their involvement in modulation of neurotransmitter release. Accordingly, S1P at a concentration of 10 nmol/L was reported both to trigger glutamate release and to potentiate depolarization-evoked glutamate secretion in cultured hippocampal neurons [42]. Moreover, application of 1 μmol/L S1P to rat hippocampal slices for 10 min was found to increase the rate of AMPA receptor-mediated miniature excitatory postsynaptic currents (mEPSCs) recorded from the CA3 region [43].

Substantial immunoreactivity against S1PR1 and S1PR3 was observed in nerve terminals outside the active zone. Notably, the extrasynaptic fraction contains presynaptic plasma membrane that is not part of the active zone, as well as membranes from intracellular organelles (depicted in Fig. 1A) [44, 45]. Therefore, receptors in this fraction comprise pools located within intracellular membranes, and can be readily trafficked to the active zone on demand, as proposed for S1PR3 [43, 46]. The present results show that only S1PR2 and S1PR3 are placed within the postsynaptic density where a marked enrichment of *N*-methyl-D-aspartate (NMDA) receptor subunits is expected [44, 47, 48]. This observation provides molecular support for a role of S1PR2/3 signalling in controlling NMDA receptor function, and therefore participating in synaptic plasticity. The importance of S1P signalling in synaptic plasticity has been evaluated by studies showing S1P-dependence of mossy fiber-CA3 long-term potentiation in hippocampal slices from mice that lack the S1P generating enzyme Sphingosine kinase 1 (SphK1) [43]. Similarly, *Caenorhabditis elegans* mutants lacking SphK1 presented impaired neurotransmitter release from neuromuscular junctions [49]. S1P-mediated modulation of hippocampal long-term potentiation was suggested to involve pre-synaptic S1PR3-mediated controlling of glutamatergic transmission [43, 46]. Although our results provide evidence for S1PR3 located mainly extrasynaptically, it does not exclude its presence in the active zone (Fig. 1F).

Exogenous S1P application led to a dose-dependent reduction in the number of responding neurons without apparent effects on the mean frequency or mean amplitude of calcium transients in our study. This lack of calcium transient modulation in active neurons upon exogenous S1P treatment is likely due to simultaneous binding to the 5 receptors that show distinct cellular locations (see Fig. 1), and might also be assigned to specific receptor kinetics or their targeting to diverse intracellular signalling pathways [50]. In addition to effects mediated through its receptors, S1P is also a notable intracellular second messenger that directly modulates Ca^2+^ homeostasis [51, 52]. However, exogenously applied S1P is taken up by cells at a rate that can be considered negligible within the few min of our calcium imaging experiments [53–55]. Therefore, we can disregard intracellular effects of exogenously applied S1P in the time course of our experiments in cultured neurons. In turn, stimulation of individual S1PRs with specific agonists revealed apparent S1PR1, 2 and 4-mediated effects on frequency and/or amplitude of calcium transients. While S1PR1, 2 and 4 dampened the amplitude or inhibited the frequency of the calcium transients, only S1PR2 modulated the fraction of responding neurons. Such discrepancy cannot be solely attributed to sub-cellular distribution of receptors since S1PR2 and 4 show identical synaptic localization. Other factors contributing to distinct features of S1PR stimulation might include the presence of co-regulatory agents, and the heterotrimeric G-proteins that are recruited [56].

Thus far, S1PR5 abundance has been mainly discussed in oligodendrocytes [21]. Our data using immunoblotting and immunofluorescence with a validated antibody [57] suggests that it is present in neurons and their synapses. S1PR5 localization showed distribution patterns in synapses similar to those of S1PR1. However, while the S1PR1 agonist affected the amplitude of calcium transients in cultured neurons, S1PR5 activation was devoid of any significant effects. It is likely that S1PR5 levels in neurons and synapses are much lower than those of other receptors, which could be quantitatively ascertained by binding experiments using labeled selective agonists. Notably, in the mouse cortex, immunofluorescence microscopy did not show detectable S1PR5 immunoreactivity in all neurons, in contrast to other S1PRs (see Fig. 2A).

This study did not explore S1PRs in different neuronal populations, although it is believed that modulation of activity by S1P might occur preferentially in excitatory neurons. Riganti et al. [15] showed that S1P applied to rat hippocampal neurons at 50 nmol/L for 30 min mobilizes synapsin I from synaptic vesicles to extrasynaptic areas in glutamatergic but not GABAergic synapses. Synapsin I is a presynaptic phosphoprotein that controls the availability of synaptic vesicles for exocytosis, and thus can mediate S1P effects on glutamate release. Given the presynaptic location of S1PR2/4 found in this study, they could strategically operate S1P-dependent glutamatergic regulation.

The studies discussed above poise brain S1P as a physiological modulator of synaptic activity, and the present work supports the view of a S1P neuromodulatory system with highly heterogeneous distribution of its membrane receptors within cortical synapses. While acting as neuromodulator at relatively low concentrations [15, 42, 43, 49], S1P becomes neurotoxic at concentrations of 10 µmol/L and above [9, 58]. Exacerbated production of S1P might occur in disease (e.g. [3, 4]), and then contribute to dampening neurotransmission, synaptic plasticity, and overall depression of neuronal activity. In such conditions, selective targeting of S1PRs that are synaptically active, could constitute a strategy for improving brain function.

In conclusion, the present results obtained from fractionation of nerve terminals and spontaneous activity measurements in primary neurons has enabled us to determine that S1PR2 and S1PR4 are located in the active zone of nerve terminals, a strategic location for modulating neurotransmission, and inhibition of neuronal activity. Further studies need to explore whether and how S1PRs mediate modulation of stimulation-induced neurotransmitter release.

## Data Availability

All data generated and analyzed for the current study are included in the manuscript, and are available from the corresponding author on reasonable request.

## Abbreviations

BSA: Bovine serum albumin
CNS: Central nervous system
DMSO: Dimethylsulfoxide
FWHM: Full-width at half-maximum
NMDA: *N*-Methyl-D-aspartate
PBS: Phosphate-buffered saline
PEG: Polyethyleneglycol
PSD95: Post-synaptic density protein 95
S1P: Sphingosine-1-phosphate
S1PR: Sphingosine-1-phosphate receptor
SNAP25: Synaptosomal-associated protein 25
SphK1: Sphingosine kinase 1
